# A7 CANNABINOID 1 AND 2 RECEPTOR AGONISTS AND MU-OPIOID RECEPTOR AGONISTS SYNERGISTICALLY INHIBIT COLONIC NOCICEPTION DURING ACUTE COLITIS

**DOI:** 10.1093/jcag/gwad061.007

**Published:** 2024-02-14

**Authors:** Q K Tsang, A E Lomax, S Vanner, D E Reed

**Affiliations:** Queen's University, Kingston, ON, Canada; Queen's University, Kingston, ON, Canada; Queen's University, Kingston, ON, Canada; Queen's University, Kingston, ON, Canada

## Abstract

**Background:**

Abdominal pain is a debilitating symptom in patients with inflammatory bowel disease. Previously we have shown that combining sub-analgesic doses of cannabinoid 1 receptor (CB1R), but not cannabinoid 2 receptor (CB2R), and mu-opioid receptor (MOR) agonists synergistically inhibits colonic nociception in healthy mice. However, it is unknown whether this combination has analgesic efficacy in a pre-clinical model of colitis.

**Aims:**

To determine the effects of combining sub-analgesic doses of CBR and MOR agonists on colonic nociception during acute colitis.

**Methods:**

Colitis was induced in male and female C57BL/6 mice with 2.5% dextran sulfate sodium in drinking water. Extracellular afferent nerve recordings were obtained from *ex vivo* flat sheet preparations of mouse distal colon. Mechanosensitivity of single afferent axons was assessed via probing of the colon with a 1g von Frey hair before and after superfusion of agonists of CB1R or CB2R plus MOR. To examine effects *in vivo*, visceromotor response (VMR) to colorectal distention (volume range 20-80 µL) was measured via electromyography. Mice were injected intraperitoneally with vehicle, or a combination of CB1R or CB2R agonist plus morphine 30-minutes prior to VMR experiment. Data were analyzed using a one or two-way ANOVA with Bonferroni test. N denotes number of mice; n denotes number of single afferent axons.

**Results:**

In afferent nerve recordings, in contrast to healthy mice, the CB2R agonist HU-308 (1 µM and 3 µM) inhibited colonic mechanosensitivity in mice with colitis (1 µM: p<0.01, N=5, n=12; 3 µM: p<0.01, N=7, n=10); a lower concentration (300 nM) had no effect (p=0.52, N=6, n=10). The CB1R agonist ACEA (10 µM) reduced mechanosensitivity during acute colitis (p<0.05, n=11, N=6), whereas 100 nM (p>0.99, n=7, N=5) and 1 µM (p=0.25, n=10, N=6) had no effect. A combination of sub-analgesic concentrations of ACEA (100 nM) and DAMGO (MOR agonist, 1 nM) inhibited colonic mechanosensitivity in healthy mice (p<0.01, N=4, n=8) and during acute colitis (p<0.05, N=6, n=8). While a combination of sub-analgesic concentrations of HU-308 (300 nM) and DAMGO (1 nM) had no effect in healthy mice (p=0.70, N=4, n=8), it inhibited colonic mechanosensitivity during acute colitis (p<0.01, N=8, n=15). In VMR experiments, a combination of a sub-analgesic dose of ACEA (0.3 mg/kg) with morphine (0.3 mg/kg) reduced VMR (p<0.01, N=7) during acute colitis. Similarly, a combination of a sub-analgesic dose of HU-308 (1 mg/kg) and morphine (0.3 mg/kg) reduced VMR (p<0.01, N=6) during acute colitis.

**Conclusions:**

A CB2R agonist inhibits colonic nociception during acute colitis, but not in healthy mice. A sub-analgesic combination of CB1R and MOR agonists can inhibit pain in healthy and inflamed mice, while combining sub-analgesic CB2R and MOR agonists is only inhibitory during acute colitis.

**Funding Agencies:**

NRC

